# PGCLCs of human 45,XO reveal pathogenetic pathways of neurocognitive and psychosocial disorders

**DOI:** 10.1186/s13578-022-00925-0

**Published:** 2022-12-01

**Authors:** Dantong Shang, Tian Lan, Yue Wang, Xuanyu Li, Quanyi Liu, Huimin Dong, Bo Xu, Hanhua Cheng, Rongjia Zhou

**Affiliations:** 1 Hubei Key Laboratory of Cell Homeostasis, College of Life Sciences, Renmin Hospital of Wuhan University, Wuhan University, Wuhan, 430072 China; 2 Bio-X Institutes, Key Laboratory for the Genetics of Developmental and Neuropsychiatric Disorders, Ministry of Education, Shanghai Jiao Tong University, Shanghai, 200240 China

**Keywords:** Cognition, Psychosocial disorder, Neurodegenerative diseases, Turner syndrome, hPGCs

## Abstract

**Background:**

Neurocognitive disorders and psychosocial difficulties are common in patients with Turner syndrome and multiple neurodegenerative diseases, yet there is no effective cure. Human primordial germ cells (hPGCs) are pluripotent germline stem cells in early embryo, which pass genetic information from one generation to the next, whereas all somatic cells will die along with the end of life. However, it is not known whether patient hPGCs with Turner syndrome contain information of neurocognitive and psychosocial illness.

**Results:**

In this report, we used a high-density of culture system of embryoids derived from iPSCs of a patient with Turner syndrome to ask how pathogenetic pathways are associated with onset of neurocognitive and psychosocial disorders. The hPGC-Like Cells (hPGCLCs) were in vitro specified from iPSCs of 45,XO, 46,XX and 46,XY by the high-density induction of embryoids. Amazingly, we found that the specification process of the hPGCLCs in 45,XO, compared to those in 46,XX and 46,XY, enriched several common pathogenetic pathways regulating neurocognitive and psychosocial disorders, that shared among multiple neurodegenerative diseases and Turner syndrome. The downregulated chemical synaptic transmission pathways, including glutamatergic, GABAergic, and nicotine cholinergic synapses, indicated synaptic dysfunctions, while upregulated pathways that were associated with imbalance of mitochondrial respiratory chain complexes and apoptosis, may contribute to neuronal dysfunctions. Notably, downregulation of three types of ubiquitin ligases E1-E2-E3 and lysosome-associated sulfatases and RAB9A, owing to haploinsufficiency and parental preference of the X chromosome expression, indicated that two pathways of cellular degradation, lysosome and ubiquitin–proteasome, were impaired in the specification process of 45,XO hPGCLCs. This would lead to accumulation of undesired proteins and aggregates, which is a typically pathological hallmark in neurodegenerative diseases.

**Conclusions:**

Our data suggest that the specification process of the hPGCLCs in 45,XO, compared to those in 46,XX and 46,XY, enriched pathogenetic pathways that are associated with the onset of neurocognitive and psychosocial disorders.

**Supplementary Information:**

The online version contains supplementary material available at 10.1186/s13578-022-00925-0.

## Introduction

Turner syndrome is a complex genetic disorder that approximately affects 50 in 100,000 female newborns worldwide [1, 2]. Actual prevalence of Turner syndrome is unknown, because of a high rate of spontaneous abortions of the fetuses with Turner syndrome [3–5]. As the most prevalent sex chromosome abnormality in females, the patients with Turner syndrome have only one intact X chromosome, but are completely or partially lack of the second sex chromosome, and over half of them are 45,XO [6]. Abnormalities in Turner syndrome involve multiple organ systems through all stages of life, and main clinic features include short stature, neck webbing, hand and foot anomalies, infertility, congenital heart disease, renal abnormalities, hearing impairment, type 2 diabetes mellitus, autoimmunity, and intrauterine lethality, in addition to neurocognitive and psychosocial disorders [2, 7–11]. Because of the complex dysfunctions involved in multiple systems, the underlying pathogenetic mechanisms of Turner syndrome have not been elucidated well, in particular, in neurocognitive and psychosocial problems.

Girls and women with Turner syndrome frequently experience neurocognitive and psychosocial health issues, mainly including intellectual disability, depressive or anxiety disorder, autism, and schizophrenia. In intellectual disability, an eightfold increased risk of mental retardation was observed in girls and women with Turner syndrome compared with age-matched controls [12]. Previous study showed thirteen percent of the 111 girls with Turner syndrome were diagnosed with mental illness [6]. With regards to depressive or anxiety disorder, forty-two percent of patients with Turner syndrome met criteria for current or past depressive or anxiety disorders during their lifetime, 48% for a past Axis I psychiatric illness, and 18% for a current Axis I psychiatric disorder [13], and 32% reported anxiety and/or depression [14]. Indeed, adolescents and adults with Turner syndrome experienced frequent and severe depressive symptoms, and adulthood are at higher risk for depression [10]. In phenotypes of autism and schizophrenia, a large cohort study showed a fourfold increased risk for autism spectrum disorders and twice the risk for schizophrenia and related disorders [12]. In addition, specific cognitional and psychosocial deficiencies are also frequently detected in patients with Turner syndrome and mainly include difficulties in understanding mathematics, defective space-form perception or visual-spatial deficits, problems in speed and fluency of lexigraphic association, facial expression recognition and body image, defective motor coordination, working memory, executive control, attentional control, self-esteem, social cognition, immaturity and attention deficits, and behavioral and emotional disorders [2, 3, 7, 11, 12, 14–16]. However, molecular mechanisms underlying onset of these neurocognitive and psychosocial disorders in Turner syndrome remain largely unknown.

Neurodegenerative diseases are characterized by progressive neuronal dysfunction and death, which are common in people aged 60 and older. Worldwide, the prevalence of these disorders is increasing and approximately 50 million people have dementia and the number will increase to 82 million in 2030 [17, 18]. In clinical manifestations, it is noteworthy that neurocognitive and psychosocial profiles in Turner syndrome are often overlapped with clinical features of multiple neurodegenerative disorders, including Alzheimer’s disease, Parkinson’s disease, Huntington’s disease, and other related diseases. For example, Alzheimer’s disease is featured by dementia, memory loss, visuospatial dysfunction, impaired language functions, agitation, psychosis, delusions and hallucinations, depression and apathy, sleep disorders, alterations in personality and behavior [19, 20]. Parkinson’s disease is characterized by progressive death of dopaminergic neurons, leading to motor disfunction and also nonmotor impairment, including dementia, psychosis, autonomic disturbances, illusions, delusions and hallucinations, anxiety, depression and apathy, sleep disorders, cognitive impairment, visuospatial dysfunction, executive functioning deficits [21, 22]. It is generally believed that aggregates, deposits, and inclusions of misfolded or damaged proteins in brain, are main contributors of these neurodegenerative diseases [23], although the underlying molecular mechanisms remain elusive.

Because Turner syndrome is associated with a complete or partial loss of the second sex chromosome, a great effort has been put into assessing effects of the genes on the X chromosome that are potentially implicated in Turner syndrome. Growing evidence shows that the retained X chromosome in Turner syndrome is maternal in origin in 70–80% cases and the paternal X or Y prefers to be lost [3, 5, 24]. This is consistent with the fact that the 45,Y liveborns are seldomly observed [25]. With regard to the parental origin of the X chromosome, a putative X-linked imprinting effect was observed on both cortical thickness and volume in Turner syndrome [26] and cognitive function [27, 28]. Nevertheless, the significant X-linked imprinting effects were not detected, although an increased prevalence of attention-deficit/hyperactivity disorder in patients with Turner syndrome [29]. Sociocognitive phenotype in Turner syndrome was also not associated with the X-linked genomic imprinting [30]. However, the X chromosome instability was associated with a globally reduced methylation level in embryonic stem cell lines [31]. Haploinsufficiency on the X chromosome could potentially contribute to the phenotypes of Turner syndrome. By far, haploinsufficiency of the escape gene SHOX on chromosome X is convincingly linked to short stature of Turner syndrome [2, 32]. The escape gene KDM5C on the X chromosome showed an association with neurocognitive profiles of patients with Turner syndrome [33]. KDM5C is a histone demethylase responsible for transcriptional repression of neuronal genes [34]. Kdm5C KO mice show multiple neurodevelopmental disorders including cognitive abnormalities and memory deficits [35]. In addition, the deletion of Xp22.3 region is responsible for a neurocognitive phenotype of patients with Turner syndrome [36]. Although these efforts, key genes and related pathways for neurocognitive and psychosocial disorders in Turner syndrome remain unknown. Moreover, the pathogenetic mechanisms of multiple neurodegenerative disorders also remain elusive. Given a shared clinic feature in neurocognitive and psychosocial problems among Turner syndrome and multiple neurodegenerative disorders including Alzheimer’s disease, Parkinson’s disease, Huntington’s disease, and other related diseases, it captures our attention to uncover their common genes, pathways, and underlying potential pathogenetic mechanisms.

Human primordial germ cells (hPGCs) are pluripotent germline stem cells in early embryo. Based on the germ-plasma theory of heredity proposed by early biologist August Weismann [37], germline cells pass both genetic and epigenetic information from one generation to the next, in contrast, all somatic cells will die along with the end of individual life [38]. Thus, hPGCs store all information of both health and disease in humans. In this report, we used a high-density of culture system of embryoids derived from the induced pluripotent stem cells (iPSCs), to explore the underlying pathogenetic mechanisms of the neurocognitive and psychosocial disorders. hPGC-Like Cells (hPGCLCs) were in vitro specified from the iPSCs derived from patient (45,XO) with Turner syndrome through induction of embryoids. In surprise, we found that the specification process of hPGCLCs of 45,XO, compared to those of both 46,XX and 46,XY, enriched common pathological pathways regulating neurocognitive and psychosocial disorders that shared among multiple neurodegenerative diseases and Turner syndrome. In addition to recapitulating pathogenetic features of Turner syndrome, our findings presented several pathogenetic pathways, and elucidated potential mechanisms shared among these neurocognitive and psychosocial diseases. These data suggested that the 45,XO hPGCs contain information for later onset of the neurocognitive and psychosocial problems, and provided new strategies for clinical diagnosis and potential therapeutic development against these diseases.

## Results

### Generation of Turner embryoids from hiPSCs

**Fig. 1:**
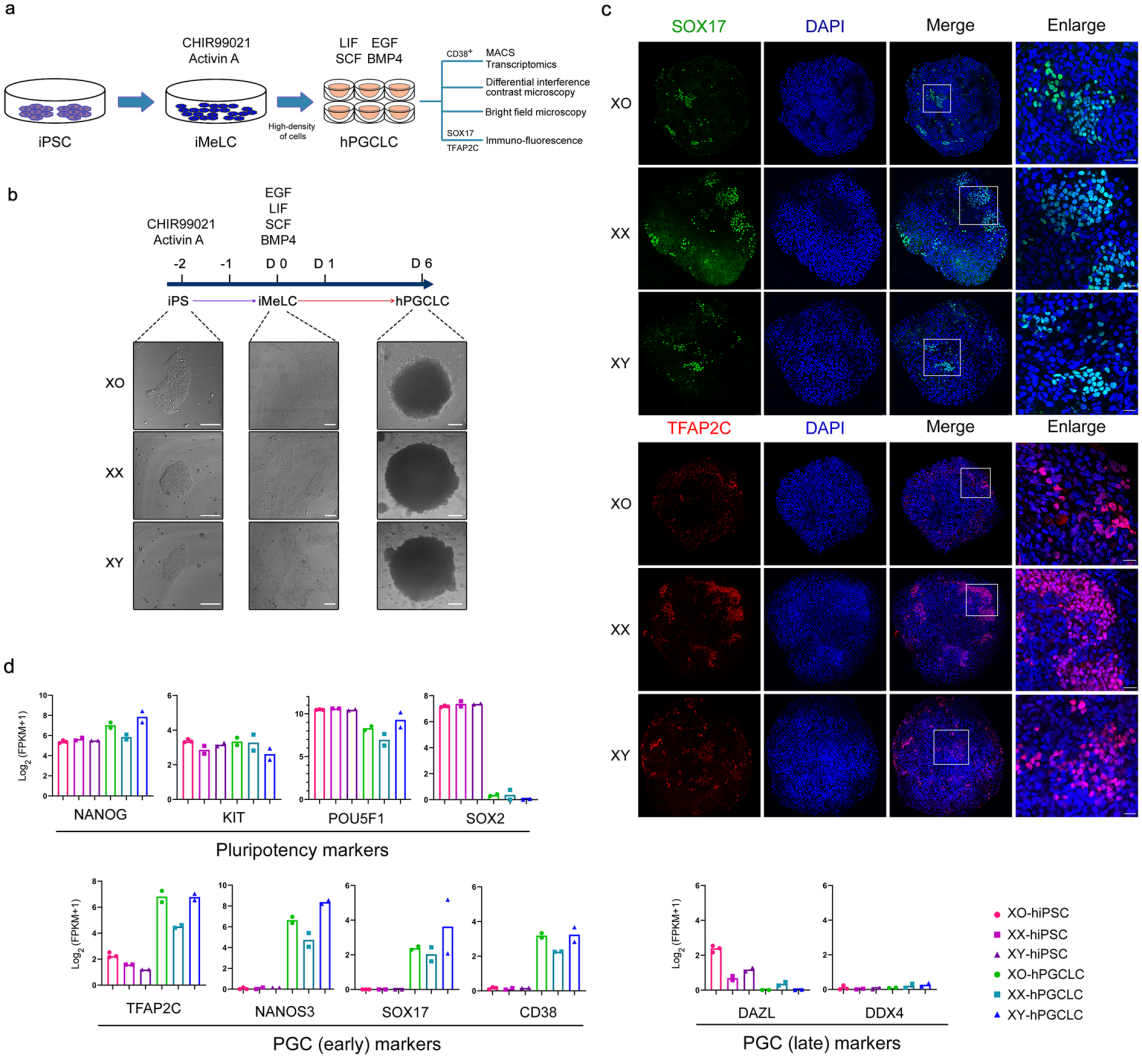
Embryoids generation and characterization from hiPSCs of 45,XO, 46,XX, and 46,XY. **a** Schematic generation of embryoids from hiPSCs via iMeLCs by high-density induction. **b** The images of hiPSCs (DIC, bar, 100 μm), iMeLCs (DIC, bar, 100 μm), and embryoids (bright field, bar, 200 μm). **c** Immunofluorescence of SOX17 (green) and TFAP2C (red) expression in day 6 embryoids. Inner sets with white line were enlarged. Images were captured with confocal microscopy. The nuclei were counterstained with DAPI (blue). Bar, 25 μm. **d** Expression of marker genes of pluripotency, hPGC (early), and hPGC (late). Y-axis indicates expression levels (Log_2_ (FPKM + 1))

To establish in vitro pathological model of Turner syndrome, hPGCLCs were induced from hiPSCs derived from the patient with Turner syndrome (45,XO) via an incipient mesoderm-like cell state (iMeLCs) by high-density of culture to form Turner embryoids (Fig. 1a). Phenotypic characteristics of the day 6 embryoids observed by differential interference contrast microscopy and bright field microscopy showed a transition of stem cell clones to flat epithelial cell state with distinct cell-to-cell boundaries and final toward embryoids of bowl shape (Fig. 1b). Phenotypically, no obvious difference was observed in these embryoids among 45,XO, 46,XX, and 46,XY. Turner embryoids (45,XO) as well as those of 46,XX and 46,XY showed obvious expression of PGC markers, SOX17 and TFAP2C, in their nuclei (Fig. 1c). In addition, other early PGC marker genes and pluripotency marker genes except SOX2 were expressed in hPGCLCs isolated from embryoids by MACS by CD38, a hPGCs surface marker, and late PGC marker genes were not expressed in hPGCLCs, whereas pluripotency marker genes including SOX2 were expressed in hiPSCs (Fig. 1d). These data revealed a Turner embryoid model containing hPGCLCs derived from the hiPSCs of the patient with Turner syndrome.

### Common pathogenetic pathways regulating Turner syndrome and neurodegenerative diseases

**Fig. 2:**
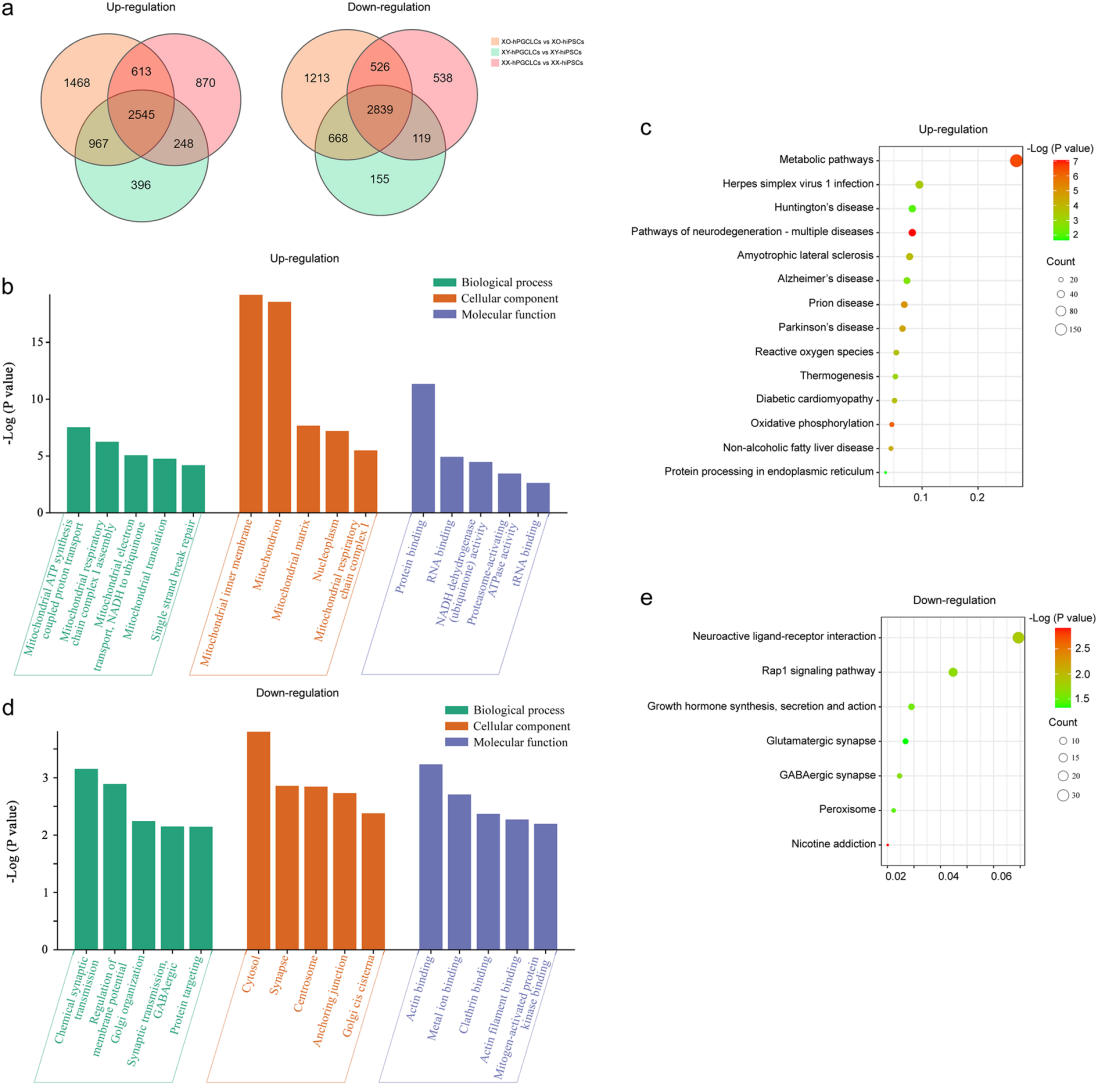
GO and KEGG analysis of differential expression genes (DEGs) in 45,XO embryoids during hPGCLC differentiation from hiPSCs. **a** Venn diagram showing number of DEGs (both up- and down-regulation) in hPGCLCs from embryoids during specification among 45,XO, 46,XX, and 46,XY. **b** Gene Ontology (GO) analysis of up-regulated DEGs among 45,XO, 46,XX, and 46,XY. X-axis indicated the catalogs of biological process, cellular component, and molecular function. Y-axis on the left indicated the -Log (P value). **c** KEGG analysis of up-regulated DEGs. The cycle sizes represented the actual number of DEGs that were classified in a particular pathway. X-axis indicated the ratio of genes in the particular pathway. **d** GO analysis of down-regulated DEGs. X-axis indicated the catalogs of biological process, cellular component, and molecular function. Y-axis indicated the -Log (P value). **e** KEGG analysis of down-regulated DEGs

To demonstrate that the embryoid model recapitulates pathogenetic processes of Turner syndrome and neurodegenerative diseases, transcriptomes of both hiPSCs and the corresponding CD38^+^ hPGCLCs of three karyotypes, 45,XO, 46,XX, and 46,XY, were analyzed to identify differential expressed genes (DEGs) in 45,XO during the specification process of hPGCLCs derived from hiPSCs (Fig. 2a). CD38 is a surface marker expressed in hPGCLCs [39], which was used to isolate CD38^+^ hPGCLCs from the embryoids by MACS technology using anti-CD38 (Fig. 1a). Gene Ontology (GO) and Kyoto Encyclopedia of Genes and Genomes (KEGG) analysis were used to identify molecular pathways of DEGs involved in the specification process in hPGCLCs of 45,XO, compared to those in both 46,XX and 46,XY. Up-regulated GO terms in 45,XO mainly included mitochondrial functions and energy metabolism (Fig. 2b). Interestingly, the related up-regulated KEGG pathways were involved in multiple neurodegenerative diseases (Alzheimer’s disease, Parkinson’s disease, and Huntington’s disease) (Fig. 2c). Moreover, down-regulated GO terms mainly included chemical synaptic transmission and synapse function (Fig. 2d), and the related down-regulated KEGG pathways were neuroactive ligand-receptor interaction, Rap1 signaling pathway, growth hormone synthesis, secretion and action, glutamatergic synapse, GABAergic synapse, and nicotine addiction (Fig. 2e). These results suggested that the Turner embryoids stored common pathogenetic pathways regulating multiple disorders in neurodegenerative diseases, in addition to Turner syndrome.

### Growth hormone action and RAP1 signaling for short stature

Short stature is one of main clinical manifestations of Turner syndrome. The KEGG analysis of DEGs showed that the key pathway regulating growth hormone (GH) synthesis, secretion and action was enriched in the specification process of hPGCLCs of 45,XO, compared to those of both 46,XX and 46,XY, in particular, key genes for GH synthesis, secretion and action were significantly down-regulated (Additional file 1: Fig. S1a). Differential expression analysis showed that main genes in the pathway were markedly down-regulated in 45,XO-hPGCLCs, including CREB3L4, PLCB4, and SHC3 (Additional file 1: Fig. S1b, c). These data suggested that impaired GH pathways were associated with short stature.

Moreover, RAP1 signaling pathway was enriched during the specification process of hPGCLCs of 45,XO, compared to those of both 46,XX and 46,XY (Additional file 1: Fig. S2a). RAP1 is a GTPase with inactive or active state through the corresponding GDP/GTP switch, in which key site at amino acid 12 (G to V) in RAS domain is important for RAP1 activation (Additional file 1: Fig. S2b). Active RAP1 coupled secondary messengers-Ca2^+^ and diacylglycerol (DAG) [40], which regulated cell proliferation, adhesion, migration, and polarity via VAV2, KRIT1, RGS14, RASSF5, and MAPSK3. The pathway related to growth factors (FGF4, FGF22, and PDGFD), RTK, and PLCB4 was also associated with visual processing defects, ataxia, absence seizures, epilepsy, and Huntington's disease [41]. Differential expression analysis showed that main genes in the RAP1 signaling pathway were markedly down-regulated in the specification process of hPGCLCs of 45,XO, including FGF4, FLT4, GRIN1, LAT, VAV2, KRIT1, RGS14, RASSF5, and MAPSK3 (Additional file 1: Fig. S2c, d), which influenced excitability, synaptic plasticity, learning, and memory [42], in addition to cellular proliferation, growth, and development [43]. These data suggested that the enriched RAP1 signaling was probably involved in regulation of neuronal plasticity, cognition function, and short stature.

### Impaired chemical synaptic transmission for synaptic dysfunctions

**Fig. 3:**
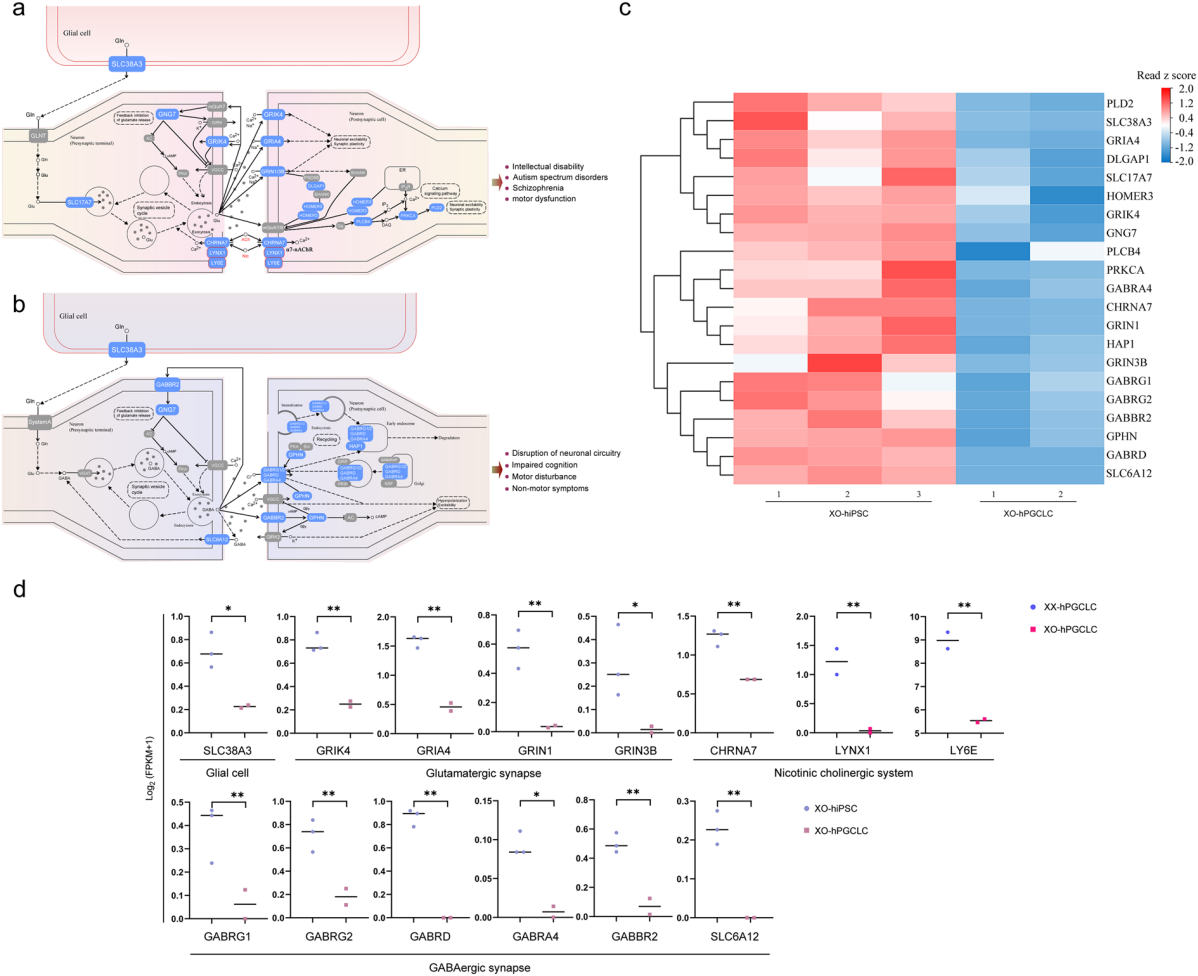
Molecular pathways regulating the transmission of glutamate, GABA, acetylcholine, and nicotine in Glutamatergic and GABAergic synapses, and nicotinic cholinergic system involved in Turner syndrome and neurodegenerative diseases. **a** Transmission pathways of glutamate, acetylcholine, and nicotine in Glutamatergic synapse and nicotinic cholinergic system associated with intellectual disability, autism spectrum disorder, schizophrenia, motor dysfunction, and antagonistic effect of nicotine addiction. DEGs in blue squares were down-regulated in 45,XO-hPGCLCs during specification from 45,XO-hiPSCs in comparison with those of both 46,XX, and 46,XY. DEGs in red cycles were down-regulated in 45,XO-hPGCLCs compared to both 46,XX-hPGCLCs and 46,XY-hPGCLCs. **b** Transmission pathways of GABA in GABAergic synapses associated with the disruption of neuronal circuitry, impaired cognition, motor disturbance and non-motor symptoms. DEGs in blue squares were down-regulated in 45,XO-hPGCLCs during specification from 45,XO-hiPSCs in comparison with those of both 46,XX, and 46,XY. **c** Heat map of down-regulated DEGs from (a and b) in 45,XO-hiPSCs (n = 3) and 45,XO-hPGCLCs (n = 2). **d** Spot plots showing expression of representative DEGs in glutamatergic and GABAergic synapses, and nicotinic cholinergic system in 45,XO-hiPSCs (n = 3) and 45,XO-hPGCLCs (n = 2). Expression levels of LYNX1 and LY6E were detected in 45,XO-hPGCLCs (n = 2) and 46,XX-hPGCLCs (n = 2). Y-axis indicates expression levels (Log_2_ (FPKM + 1)). *P < 0.05; **P < 0.01

The shared clinic features in neurocognitive and psychosocial problems among Turner syndrome and multiple neurodegenerative diseases may imply an intrinsic molecular link. However, the molecular mechanisms underlying these shared disorders were unknown yet. As KEGG analysis indicated that nine of top 10 terms were associated with multiple neurodegenerative diseases, it is particularly interesting to identify key genes and related pathways involved in neurocognitive and psychosocial problems. KEGG analysis of DEGs showed that key genes involved in glutamatergic synapse, GABAergic synapse, and nicotine cholinergic system were significantly down-regulated in the specification process of hPGCLCs of 45,XO derived from hiPSCs, compared to those of both 46,XX and 46,XY (Fig. 3a, b). Down-regulation of these pathways in glutamatergic synapse was associated with impairment of neuronal excitability and synaptic plasticity (Fig. 3a). Abnormality of neuronal excitability and loss of synaptic plasticity often lead to intellectual disability, autism, schizophrenia and motor dysfunction [44–46]. Down-regulated pathways in GABAergic synapse included processes of generation, recycle, and transmission of GABA (Fig. 3b). Dysfunctions of GABAergic synapse caused disruption of neuronal circuitry, impaired cognition, motor disturbance, and other non-motor symptoms [47–49]. Moreover, cholinergic system was also down-regulated. Failure of cholinergic system was observed in patients with Alzheimer’s disease [50]. Dysfunctions of cholinergic system were involved in impairment of attention and memory [51]. Differential expression analysis revealed that main genes in these pathways were significantly down-regulated in 45,XO-hPGCLCs, including SLC38A3, GRIN1, GRIN3B, CHRNA7, GABRG1, and SLC6A12 (Fig. 3c, d), which were potential target genes for chemical synaptic transmission.

### Pathways of ROS and apoptosis for neuronal dysfunctions

**Fig. 4:**
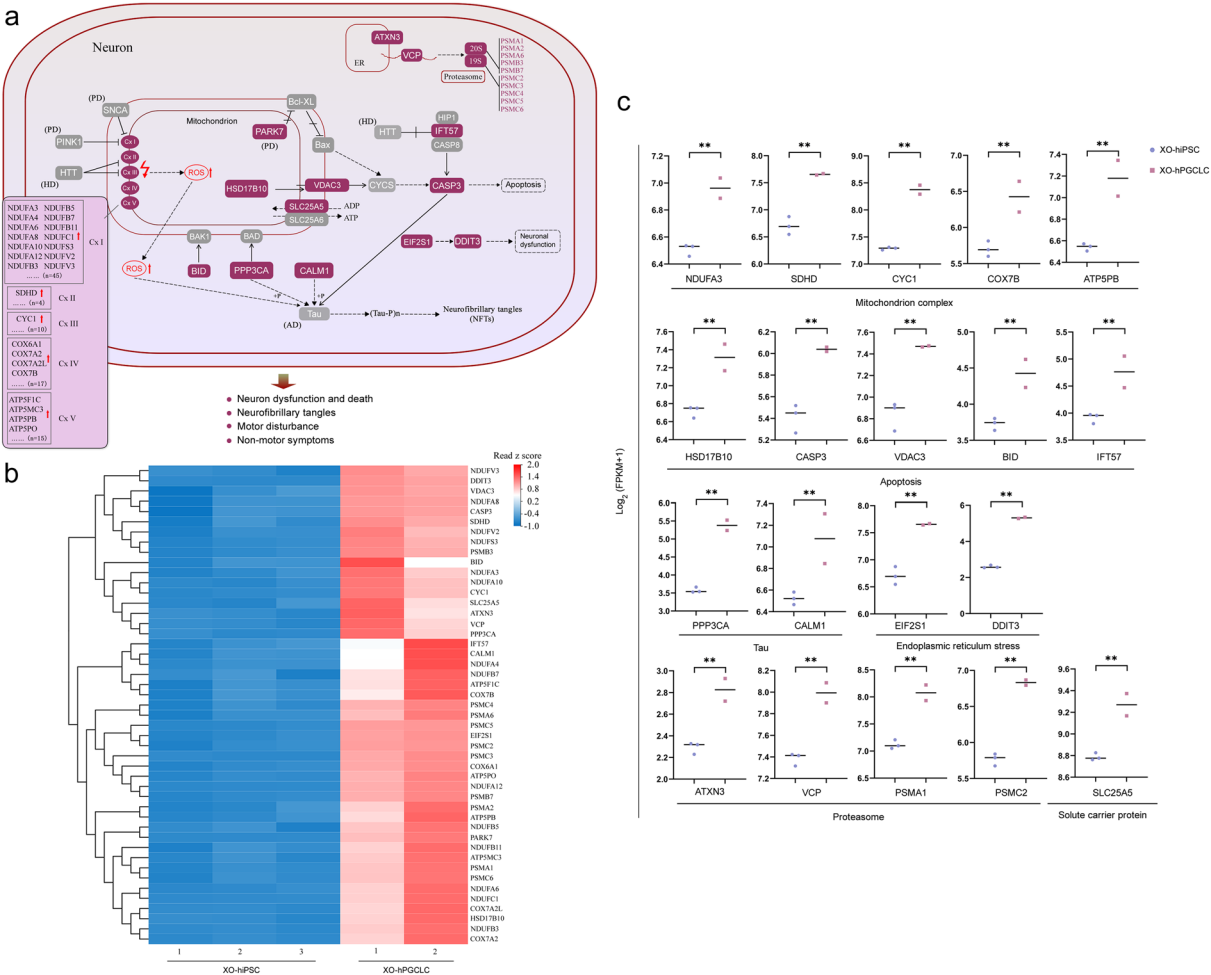
Pathways of ROS and apoptosis for neuronal dysfunctions. **a** Molecular pathways regulating oxidative damage, mitochondrial dysfunction, and apoptosis associated with neuronal dysfunction and death, neurofibrillary tangles, motor disturbance and non-motor symptoms. DEGs in red squares were up-regulated in 45,XO-hPGCLCs during specification from 45,XO-hiPSCs in comparison with those of both 46,XX, and 46,XY. Red arrows indicated up-regulation. ROS, reactive oxygen species. **b** Heat map of up-regulated DEGs from (a) in 45,XO-hiPSCs (n = 3) and 45,XO-hPGCLCs (n = 2). **c** Spot plots showing expression of representative DEGs from (a) in 45,XO-hiPSCs (n = 3) and 45,XO-hPGCLCs (n = 2). Y-axis indicates expression levels (Log_2_ (FPKM + 1)). **P < 0.01

Further analysis of DEGs showed that up-regulated pathways were mainly involved in mitochondrial electron transport/respiratory chain, VDAC3-CASP3 apoptosis, EIF2S1-DDIT3/CHOP ER stress, proteasome formation, and tau pathology (Fig. 4a). In electron transport/respiratory chain, a number of subunits in each complex (I, II, III, IV, and V) were up-regulated (Fig. 4a). Up-regulated subunits accounted for 31% in complex I, 25% in complex II, 10% in complex III, 17.6% in complex IV, and 26.7% in complex V, while the other subunits were not affected in these complexes, implying imbalanced components in each complex. Imbalance of mitochondrial respiratory chain complexes probably causes the electron leakage in respiratory chain, which led to high levels of reactive oxygen species (ROS) [52]. Caspase-3 is well known for proteolysis during apoptosis. Thus, upregulation of VDAC3-CASP3 pathway was associated with apoptosis. Meanwhile, CASP3 upregulation cleaved tau protein into truncated form of tau, which was an early event in Alzheimer’s disease tangle pathology [53] and important for the pathogenesis of Alzheimer’s disease through the impairment of mitochondrial dynamics [54]. Interestingly, both PPP3CA and CALM1 were upregulated, which were key proteins to phosphorylate tau protein. Hyperphosphorylation of tau protein was an important event for formation of neurofibrillary tangles (NFTs), along with tau truncation. NFTs were the characteristic pathogenetic hallmarks of Alzheimer’s disease [55]. Upregulation of EIF2S1-DDIT3/CHOP represents ER stress, thus leading to neuronal dysfunctions [56]. In addition, proteasome components were also up-regulated. These molecular and cellular processes were probably associated with neuronal dysfunction and death, motor disturbance, and non-motor symptoms including cognitive difficulties. Differential expression analysis revealed a couple of main up-regulated genes in these pathways in the specification process of 45,XO-hPGCLCs, compared to those of both 46,XX and 46,XY, including NDUFA3, SDHD, CYC1, COX7B, ATP5PB, CASP3, PPP3CA, CALM1, DDIT3, and VCP (Fig. 4b, c), which were important genes for neuronal functions.

### Parental preference expression of chromosome X in specification process of hPGCLCs from hiPSCs in 46,XX female

**Fig. 5:**
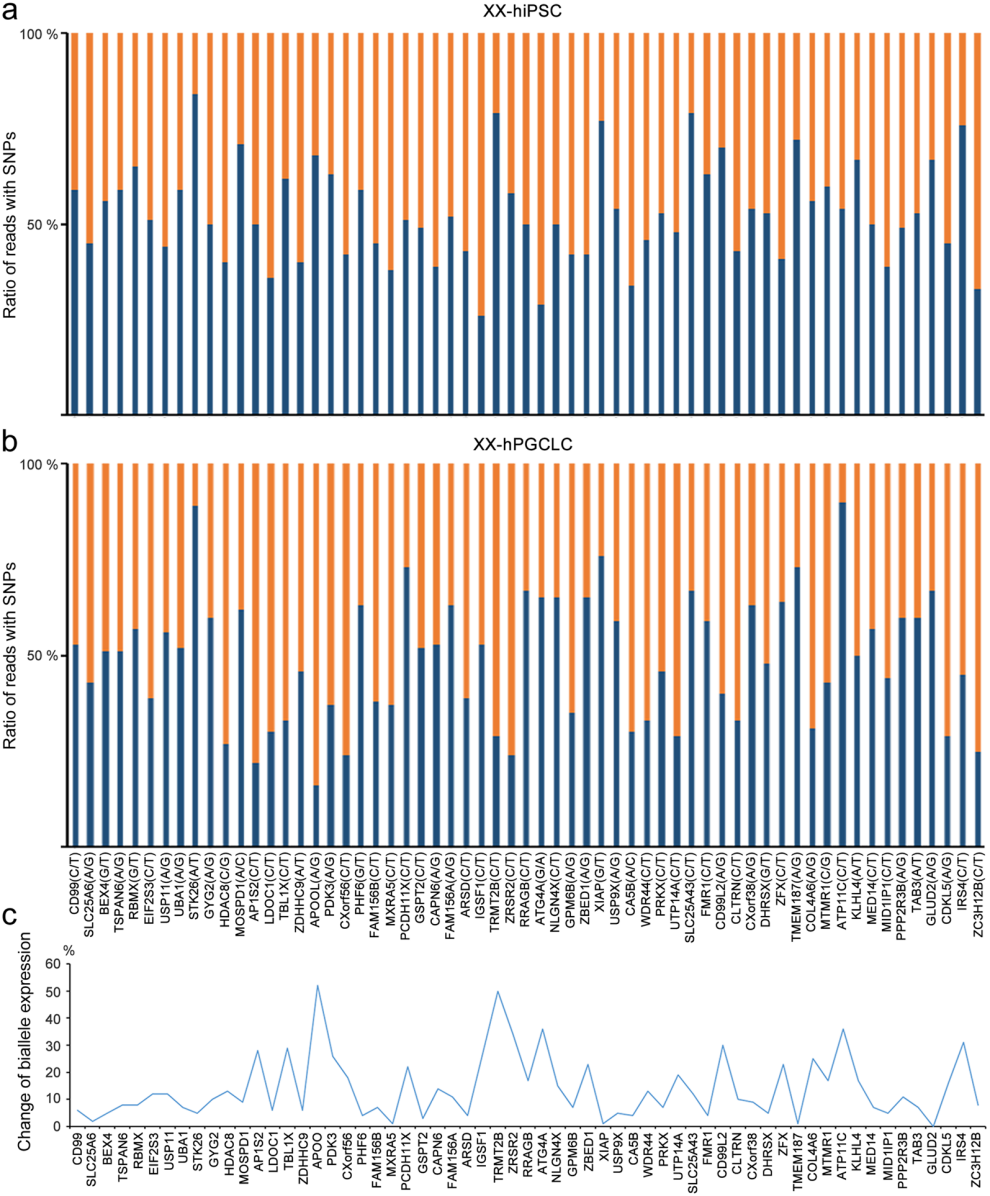
Parental preference of biallele expression of X-linked genes with shared SNPs in 46,XX-hPGCLCs compared to 46,XX-hiPSCs. **a** Ratio of reads with SNPs in X-linked bialleles in 46,XX-hiPSCs. **b** Ratio of reads with SNPs in X-linked bialleles in 46,XX-hPGCLCs. **c** Percentage change of biallele expression of X-linked genes in 46,XX-hPGCLCs compared to 46,XX-hiPSCs

To explore whether haploinsufficiency of the X-linked genes is linked with Turner syndrome and neurodegenerative diseases, all genes on X chromosome were used to comparatively analyze their differential expression in hPGCLCs from embryoids between 45,XO and 46,XX. It is interesting whether parental preference presents during specification process of hPGCLCs from hiPSCs derived from the same female with 46,XX, if so, whether it is associated with haploinsufficiency of the X-linked gene expression in 45,XO and 46,XX. Bialleles with shared SNPs on both X chromosomes were used to analyze parental preference of expression in 46,XX. Notably, ratio of the reads with SNPs in hPGCLCs obviously skewed the ratio of 50% in comparison with that (~ 50%) of hiPSCs from same individual with 46,XX (Fig. 5a, b). Percent change of biallele expression in the hPGCLCs skewed, on average, 13.93%, compared to the original hiPSCs (Fig. 5c). These data suggested that expression of bialleles in parental two X chromosomes has a preference of one parent during specification process of hPGCLCs from hiPSCs.

### Haploinsufficiency of the X genes is associated with dysfunctions of lysosome and ubiquitin mediated proteolysis

**Fig. 6:**
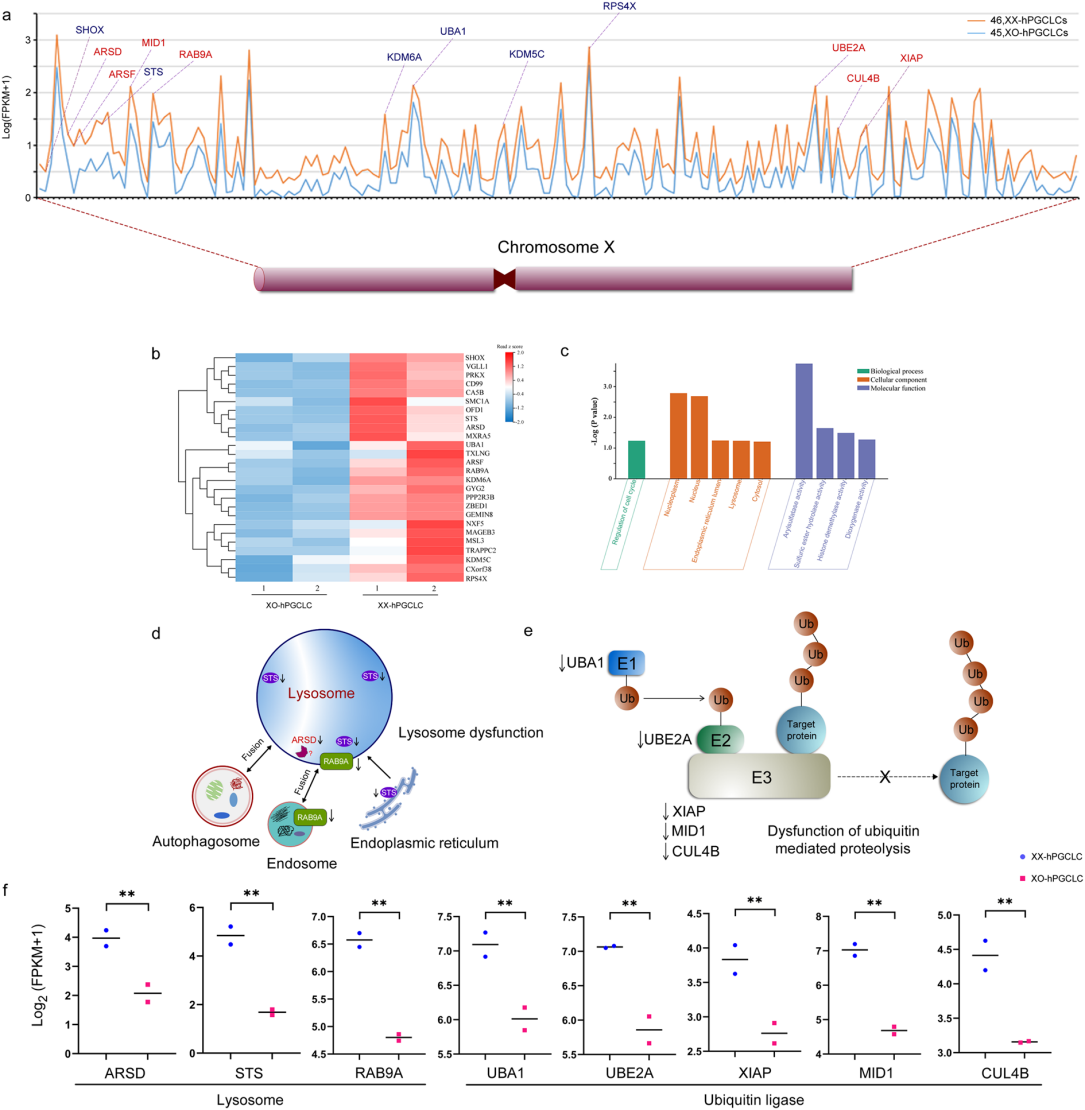
Haploinsufficiency genes on X chromosome and related pathways involved in dysfunction of lysosome and impairment of ubiquitin mediated proteolysis in 45,XO-hPGCLCs. **a** Relative expression levels of 184 haploinsufficiency genes along X chromosome between 45,XO-hPGCLCs (blue line) and 46,XX-hPGCLCs (orange line). Y-axis indicates expression levels (Log_2_ (FPKM + 1)). Genes in blue indicated the escape genes associated Turner syndrome. Genes in red represented haploinsufficiency genes involved in dysfunction of lysosome and impairment of ubiquitin mediated proteolysis. **b** Heat map of the X-linked escape genes in 45,XO-hPGCLCs (n = 2) and 46,XX-hPGCLCs (n = 2). **c** GO analysis of the escape genes. X-axis indicated the catalogs of biological process, cellular component, and molecular function. Y-axis indicated the -Log (P value). **d** Escape genes involved in lysosome dysfunction. Impairment of autophagy-lysosome and late endosome-lysosome fusion, and dysfunction of lysosome are associated with Turner syndrome and neurodegenerative diseases. **e** Haploinsufficiency genes in ubiquitination pathway involved in dysfunction of ubiquitin mediated proteolysis in Turner syndrome and neurodegenerative diseases. **f** Spot plots showing expression of representative haploinsufficiency genes in 45,XO-hPGCLCs (n = 2) and 46,XX-hPGCLCs (n = 2). Y-axis indicates expression levels (Log_2_ (FPKM + 1)). **P < 0.01

Increased evidence suggests that the retained the X chromosome in Turner syndrome is maternal in origin in 70–80% cases and the paternal X or Y prefers to be lost [3, 5, 24]. Given that haploinsufficiency of expression of X-linked genes appears in patients with Turner syndrome, differential expression levels of all genes on chromosome X between 45,XO-hPGCLCs and 46,XX-hPGCLCs were analyzed to identify DEGs. When DEGs with fold change (FC) < 0.5 (XO:XX) were defined as haploinsufficiency, 184 haploinsufficiency genes were identified, including 28 escape genes, 76 inactive genes, and 80 other genes (Fig. 6a). Heat map showed that expression levels of the escape genes were obviously down-regulated in 45,XO-hPGCLCs compared with those of 46,XX-hPGCLCs (Fig. 6b). Of the escape genes, SHOX, STS, KDM5C, KDM6A, UBA1, and RPS4X were associated with Turner syndrome [2]. Further GO analysis of these escape genes revealed that pathways associated with lysosome function and arylsulfatase activity were enriched (Fig. 6c). In lysosome function, STS, ARSD, and RAB9A were downregulated in 45,XO-hPGCLCs. RAB9A exerts roles in late endosome transition and fusion with lysosome [57, 58]. STS is a steroid sulfatase and catalyzes the dehydroepiandrosterone sulfate to the biologically active dehydroepiandrosterone, and promotes androgen synthesis [59]. STS was probably transported to lysosome via mannose 6-phosphate receptor or released from the endoplasmic reticulum to lysosomes via autophagy [60]. ARSD is a candidate arylsulfatase, one of the sulfatase family members with a conserved catalytic peptide domain. Of which, ARSA and ARSB were lysosomal enzymes to hydrolyzes sulfatide by cleaving the sulfate group [61]. ARSD was associated with Alzheimer’s disease [62], although its lysosomal location and function remain unknown. These results indicated that downregulation of these genes was associated with dysfunctions of autolysosome and endosome-lysosome fusion (Fig. 6d, f). In addition, KEGG analysis of these haploinsufficiency genes showed that the pathway of ubiquitin mediated proteolysis was enriched in 45,XO-hPGCLCs. Three types of ubiquitin ligases, including E1 (UBA1), E2 (UBE2A), and E3 (XIAP, MID1, and CUL4B), were down-regulated in the pathway (Fig. 6e, f). These data suggested that haploinsufficiency genes were probably associated with dysfunctions of lysosome and ubiquitin mediated proteolysis, although proteasome components were not down-regulated. Dysfunctions of both lysosome and ubiquitin–proteasome pathways owing to the haploinsufficiency effect probably cause intracellular accumulation of undesired proteins and their aggregates, for example, Aβ aggregates and neurofibrillary tangles, which are characteristic pathogenetic hallmarks in Alzheimer’s disease.

## Discussion

Neurodegenerative diseases are characterized by progressive neuronal dysfunction and death. Neurocognitive and psychosocial disorders are common clinical manifestations in patients with multiple neurodegenerative diseases, including Alzheimer’s disease [19, 20], Parkinson’s disease [21, 22], Huntington’ s disease [63, 64], other related diseases [23], and Turner syndrome [2, 3, 12]. However, pathogenetic mechanisms underlying these shared disorders remain unknown. Here, we used high-density culture system of embryoids derived from iPSCs of Turner syndrome with a complete loss of the second sex chromosome to investigate shared pathogenetic pathways and underlying molecular mechanisms. Our study provides an unexpected finding that the specification process of 45,XO hPGCLCs displays common pathogenetic pathways regulating neurocognitive and psychosocial disorders that shared among neurodegenerative diseases and Turner syndrome, suggesting specific networks of genes and related molecular processes involved in neurocognitive and psychosocial diseases.

Our study reveals that one of common pathogenetic mechanisms for these shared disorders is synaptic dysfunctions owing to impaired chemical synaptic transmission pathways. The pathways for three key types of synapses, glutamatergic synapse, GABAergic synapse, and nicotine cholinergic system, that are essential for chemical synaptic transmission, are down-regulated in the specification process of the hPGCLCs of 45,XO. These pathogenetic pathways are associated with the pathology of neurodegenerative diseases. Glutamatergic synapse is physiologically essential for neuronal excitability and synaptic plasticity. Abnormalities of neuronal and synaptic activities often lead to intellectual disability, autism, schizophrenia, and motor dysfunction [44–46]. Impairment of GABAergic synaptic function causes disruption of neuronal circuitry, cognition deficits, motor and other non-motor disturbance [47–49]. Loss of cholinergic system and disruption of synaptic activity and neurotransmitter release often occur in Alzheimer’s disease [50, 55]. Dysfunction of cholinergic system is also involved in impairment of attention and memory [51]. Given that glutamatergic/GABAergic/cholinergic synapses are essential for synaptic and neuronal functions, our finding suggests that potential therapy strategies via the receptors, NMDAR (GRIN1/GRIN3B), KA (GRIK4), and AMPA (GRIA4), involved in glutamatergic synapse pathway, CHRNA7 in cholinergic system, and GABAA (GABRG1/2), GABRD (GABRA4), GAT (SLC6A12), and GABAB (GABBR2) in GABAergic synaptic pathways, hold great promise to improve synaptic and neuronal dysfunctions including intellectual disability, cognitive deficits, autism, and schizophrenia in neurodegenerative diseases.

We uncover several pathogenetic pathways for neuronal dysfunctions, which are associated with potential imbalance of mitochondrial respiratory chain complexes and apoptosis. Each respiratory chain complex consists of multiple subunits, for example, 45 different subunits in complex I with a combined molecular weight of ~ 1 MDa. Dysregulation of complex assembly from subunits is associated with mitochondrial function impairment, including ATP production, ROS generation, and apoptosis. Reduction of subunits of complex I was observed in brain regions of Alzheimer's disease [65], and also associated with onset of Parkinson's disease [66]. Our study showed up to half of subunits in each complex were up-regulated, but most of the other subunits were not affected in the specification process of the hPGCLCs of 45,XO, suggesting a potential imbalance or instability of the respiratory chain complexes. The imbalance could also affect supercomplex assembly among complexes. Interactions between complexes I and III, and complex III and IV were observed, which promoted formation of supercomplexes [67], and complex I assembly into supercomplexes was also associated with ROS production [68]. Imbalance or instability of the respiratory chain complexes/supercomplexes may cause the electron leakage in the respiratory chain, which often occurs mainly in complex I and complex III, thus leading to high levels of ROS [52]. ROS attack proteins, DNA and phospholipid layer, and mainly cause mitochondrial damage, thus promoting the apoptosis of neuronal cells and the onset of Alzheimer’s disease [69]. Mitochondria-mediated oxidative stress stimulated tau hyperphosphorylation, and caused accumulation of tau aggregates [70], which is pathological hallmark in the AD brain. Moreover, the apoptosis pathway VDAC3-CASP3 was up-regulated in the specification process of the hPGCLCs of 45,XO, suggesting potential apoptosis. In addition, VDAC3 can protect mitochondria from oxidative stress-induced impairment [71]. It is possible that VDAC3 also plays a partial role in balance between pro-oxidants and antioxidants in brain, although brain has a low level of antioxidants.

Another important finding in this study is parental preference expression of chromosome X in specification process of hPGCLCs from hiPSCs in female embryoids of 46,XX. In case of one X chromosome loss in Turner syndrome of 45,XO, haploinsufficiency of the retained the X chromosome probably occurs due to the parental preference expression of chromosome X. In addition, haploinsufficiency of the escape genes is also a pathogenetic risk factor for Turner syndrome and neurodegenerative diseases. Importantly, these escape genes of differential expression are associated with pathways of lysosome function and arylsulfatase activity. In lysosome function, lysosome-associated STS and ARSD are sulfatases. STS is essential for dehydroepiandrosterone synthesis [59], while function of ARSD remains unknown. Vesicle-related RAB9A functions in late endosome transition and fusion with lysosome [57, 58]. Downregulation of these lysosome-associated genes reflects lysosome dysfunction, which is consistent with accumulation of Aβ aggregates and neurofibrillary tangles. Moreover, all three types of ubiquitin ligases in ubiquitin–proteasome pathway, including E1 (UBA1), E2 (UBE2A), and E3 (XIAP, MID1, and CUL4B), were down-regulated, although proteasome components are not down-regulated, even upregulated. These data suggested two pathways of degradation, autophagy-lysosome and ubiquitin–proteasome, are impaired in the specification process of the hPGCLCs of 45,XO, which probably lead to accumulation of undesired proteins and aggregates, a typically pathogenetic hallmark in neurodegenerative diseases.

## Conclusions

This study shows that 45,XO hPGCLCs recapitulate pathogenetic features of multiple neurocognitive and psychosocial disorders that presented later in life. Enriched gene sets and pathogenetic pathways are potential targets for clinical diagnosis and therapeutic development against these diseases.

## Methods

### Cell culture, embryoid generation, and hPGCLC induction

The hiPSCs TS1 (45,XO) were derived from amniocytes at 20th week of pregnancy [72]. KY02AO-hiPSCs (46,XX) were derived from fibroblasts of adult female obtained from Shanghai Dongfang Hospital. T-hiPSCs (46,XY) were derived from T-cells of adult male from Sunshine Lake Pharma Co., Ltd. The cells were cultured in mTeSR1-plus medium (Stemcell, 1000276) on matrigel (Corning, 354277) coated 6-well plates (Sorfa, 220100). The cells were passaged every 4 days using TrypLE^™^ Select (GIBCO, 12563-011). For induction of iMeLCs, 1.5 × 10^5^ hiPSCs were plated on 12-well plates (Sorfa, 220100) coated with matrigel in GK15 medium [GMEM (Gibco, 11710035) with 15% KnockOut™ Serum Replacement (Gibco, 10828028), 0.1 mM MEM-NEAA (Sigma, M7145-100 ml), 2 mM L-glutamine solution (Sigma, G7513-20 ml), 1 mM sodium pyruvate solution (Sigma, S8636-100 ml), and 0.1 mM β-mercaptoethanol (Solarbio, M8211)] containing 50 ng/ml of Activin A (R&D systems, 338-AC/CF), 3 mM CHIR (Selleck, CT99021), and 10 mM ROCK inhibitor (Y-27632, Wako, 030–24021). For hPGCLCs induction, 3 × 10^4^ iMeLCs were plated on a low-cell-binding U-bottom 96-well plate (Thermo, 174925) in hPGCLCs induction medium. The medium is composed of GK15 medium supplemented with 200 ng/ml of BMP4 (R&D Systems, 314-BP/CF), 1000 U/ml of human LIF (Peprotech, AF-300-05-25), 100 ng/ml of SCF (Novoprotein, C034), 50 ng/ml of EGF (Gibco, PHG0311), and 10 mM ROCK inhibitor.

### Magnetic activated cell sorting (MACS)

CD38 MicroBead Kit (Miltenyi Biotec, 130092263) was used for MACS. Briefly, the day 6 embryoids containing hPGCLCs were dissociated with 0.01% Trypsin for 6–10 min at 37 °C. Dissociated cells were resuspended in MACS buffer (PBS, 0.5% BSA, 2 mM EDTA), incubated with anti-CD38-Biotin, at 4 °C for 10 min. After washing with MACS buffer, the samples were incubated with anti-Biotin MicroBeads at 4 °C for 15 min. Cell suspension was passed onto the MS column (Miltenyi Biotec, 130042201) under magnetic field and washed the column with MACS buffer. CD38^+^ cells were recovered by firmly pushing the plunger into the column.

### Immunofluorescence analysis

The day 6 embryoids were fixed with 4% paraformaldehyde for 30 min at room temperature and permeabilized with 1% Triton X-100 (Fonsber, A1050-250 ml) in PBS for 30 min and blocked in 5% BSA overnight. The samples were incubated with human SOX17 antibody (R&D, AF1924-SP) or TFAP2C antibody (Abcam, ab218107) at 4 °C overnight. After washing with PBS, the embryoids were incubated with FITC rabbit anti-goat IgG(H + L) (ABclonal, AS0580) or Alexa Flour 555-conjugated goat anti-rabbit IgG(H + L) (ABclonal, AS024) in dark at room temperature for 1 h. The nuclei were stained with DAPI (Biosharp, BS0197-10 mg). The images were taken by a confocal fluorescence microscope (Leica, SP8).

### RNA-seq samples collection and library preparation

Total RNAs were lysed by TRIzol (Solarbio, W0250), purified by Chloroform/iso-amyl alcohol, and dissolved the RNAs with RNase-free water. The mRNA was enriched using Oligo (dT) and then broken up for cDNA library construct according to the manufacturer’s protocol.

### Transcriptome sequencing and analysis

The cDNA libraries were sequenced by DNBSEQ platform. To determine gene expression levels, RNA-seq clean reads from hiPSCs (45,XO (n = 3), 46,XX (n = 2), and 46,XY (n = 2)), and d6 hPGCLCs (45,XO (n = 2), 46,XX (n = 2), and 46,XY (n = 2)) were mapped to the reference genome GCF_000001405.39_GRCh38.p13 from NCBI and accurately mapped to the reference genes by Bowtie2 [73]. Fragments per kilo bases per million fragments (FPKM) values were calculated for each gene by RSEM [74].

### Gene ontology and pathway analysis

Differentially expressed genes (DEGs) were analyzed by DEseq2[75], and defined with fold change (FC) > 1.51 and Q value < 0.05. The Gene Ontology (GO) and Kyoto Encyclopedia of Genes and Genomes (KEGG) of the DEGs were analyzed by DAVID [76, 77]. Heat maps were constructed using pheatmap (R-package) in Dr.Tom (biosys.bgi.com).

### SNPs analysis

RNA-seq data were used for formatting of Variant Call Format files and SNPs were called referring to 1000 Genomes Project to encode SNPs. Integrative genomics viewer (version 2.13.2)[78] was used to view individual aligned reads with SNPs. Allele ratio representing a relative contribution of expression of a parental allele were calculated based on reads with shared SNP sites between XX-iPSCs and XX-hPGCLCs.

### Statistical analysis

All data were presented as means ± standard error of mean from two–three independent experiments. Statistical comparisons were made using Student’s t-test when comparing two groups. Statistics analysis was performed using GraphPad Prism 8.3.0 software package (GraphPad Software, La Jolla, USA). In all analysis, P < 0.05 was considered to be statistically significant.

## Supplementary Information

**Additional file 1: Figure S1**. Molecular pathways regulating growth hormone synthesis, secretion, and action involved in short stature in Turner syndrome. **a** Molecular pathways regulating growth hormone synthesis, secretion, and action in short stature. DEGs in blue squares were down-regulated in 45,XOhPGCLCs during specification from 45,XO-hiPSCs in comparison with those of both 46,XX, and 46,XY. **b** Heat map of down-regulated genes in GH pathway in 45,XO-hiPSCs (n=3) and 45,XOhPGCLCs (n=2). **c** Spot plots showing expression of representative genes in GH pathway in 45,XOhiPSCs (n=3) and 45,XO-hPGCLCs (n=2). Y-axis indicates expression levels (Log2 (FPKM+1)).*P < 0.05; **P < 0.01. **Figure S2**. Molecular pathways regulating RAP1 GDP-GTP switch and RAP1 GTP downstream pathways involved in Turner syndrome and neurodegenerative diseases. **a **RAP1 pathways regulating cell adhesion, migration, polarity, proliferation, survival and gene activation. DEGs in blue squares were down-regulated in 45,XO-hPGCLCs during specification from 45,XO-hiPSCs 2 in comparison with those of both 46,XX, and 46,XY. **b** The mutant G to V at site 12 is an activated form of RAP1. RAS, a conserved domain in RAS family. **c** Heat map of down-regulated genes from (a) in 45,XO-hiPSCs (n=3) and 45,XO-hPGCLCs (n=2). **d** Spot plots showing expression of representative genes in RAP1 pathway in 45,XO-hiPSCs (n=3) and 45,XO-hPGCLCs (n=2). Y-axis indicates expression levels (Log2 (FPKM+1)). *P < 0.05; **P < 0.01.

## Data Availability

The accession number for the sequence reported in this paper is NCBI SRA: PRJNA877847.
