# Supplementary material for: PGCLCs of human 45,XO reveal pathogenetic pathways of neurocognitive and psychosocial disorders
Source: Cell Biosci. 2022 Dec 1;12:194. doi: 10.1186/s13578-022-00925-0 (PMC9716775; doi:10.1186/s13578-022-00925-0)

Additional files:

fig. S1. Molecular pathways regulating growth hormone synthesis, secretion, and action involved in short stature in Turner syndrome. **a** Molecular pathways regulating growth hormone synthesis, secretion, and action in short stature. DEGs in blue squares were down-regulated in 45,XO-hPGCLCs during specification from 45,XO-hiPSCs in comparison with those of both 46,XX, and 46,XY. **b** Heat map of down-regulated genes in GH pathway in 45,XO-hiPSCs (n=3) and 45,XO-hPGCLCs (n=2). **c** Spot plots showing expression of representative genes in GH pathway in 45,XO-hiPSCs (n=3) and 45,XO-hPGCLCs (n=2). Y-axis indicates expression levels (Log$_2$ (FPKM+1)). *P < 0.05; **P < 0.01.

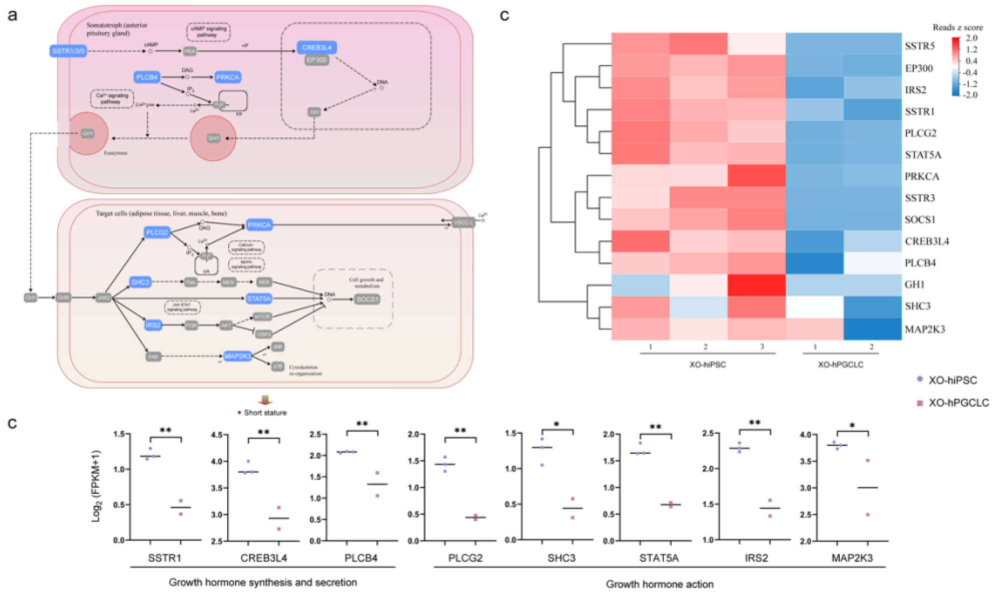

fig. S2. Molecular pathways regulating RAP1 GDP-GTP switch and RAP1 GTP downstream pathways involved in Turner syndrome and neurodegenerative diseases. **a** RAP1 pathways regulating cell adhesion, migration, polarity, proliferation, survival and gene activation. DEGs in blue squares were down-regulated in 45,XO-hPGCLCs during specification from 45,XO-hiPSCs in comparison with those of both 46,XX, and 46,XY. **b** The mutant G to V at site 12 is an activated form of RAP1. RAS, a conserved domain in RAS family. **c** Heat map of down-regulated genes from (a) in 45,XO-hiPSCs (n=3) and 45,XO-hPGCLCs (n=2). **d** Spot plots showing expression of

representative genes in RAP1 pathway in 45,XO-hiPSCs (n=3) and 45,XO-hPGCLCs (n=2). Y-axis indicates expression levels (Log_2 (FPKM+1)). *P < 0.05; **P < 0.01.

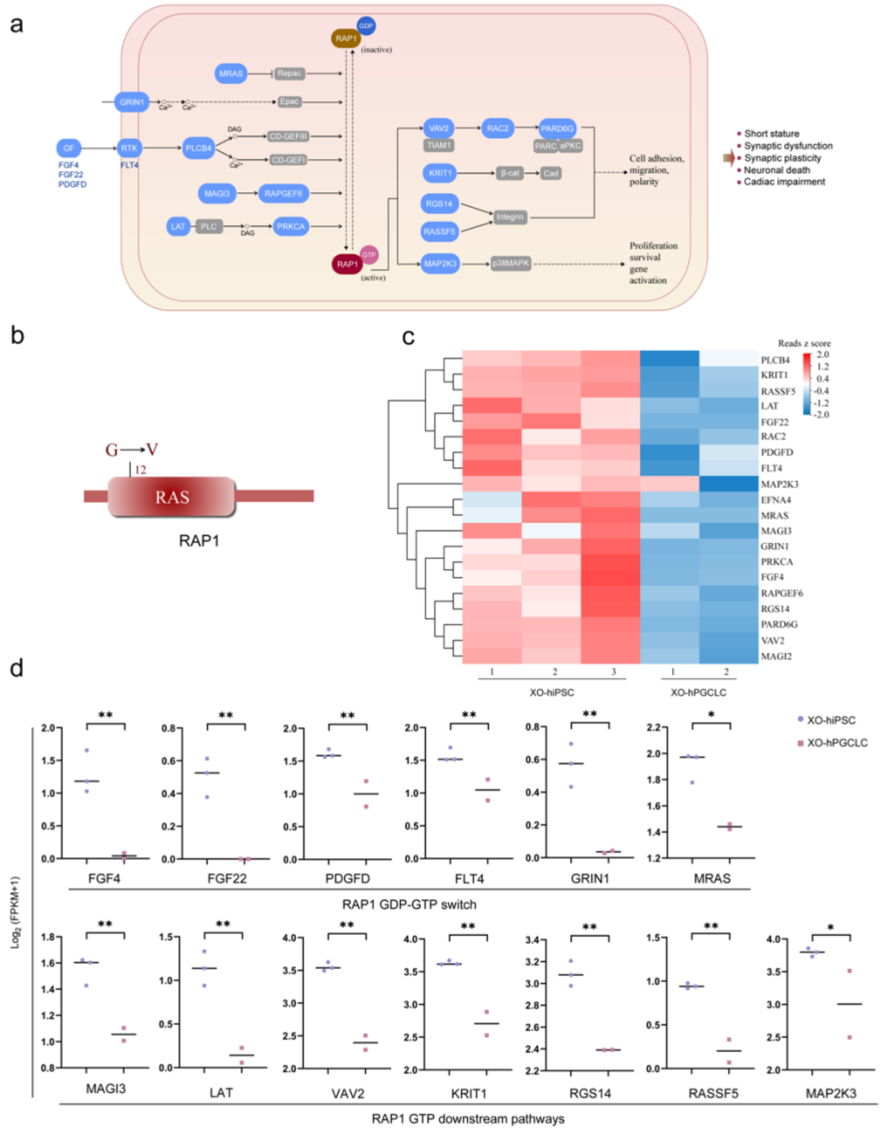

Supplement: Supplementary file 1 — Additional file 1: Figure S1. Molecular pathways regulating growth hormone synthesis, secretion, and action involved in short stature in Turner syndrome. a Molecular pathways regulating growth hormone synthesis, secretion, and action in short stature. DEGs in blue squares were down-regulated in 45,XOhPGCLCs during specification from 45,XO-hiPSCs in comparison with those of both 46,XX, and 46,XY. b Heat map of down-regulated genes in GH pathway in 45,XO-hiPSCs (n=3) and 45,XOhPGCLCs (n=2). c Spot plots showing expression of representative genes in GH pathway in 45,XOhiPSCs (n=3) and 45,XO-hPGCLCs (n=2). Y-axis indicates expression levels (Log2 (FPKM+1)).*P < 0.05; **P < 0.01. Figure S2. Molecular pathways regulating RAP1 GDP-GTP switch and RAP1 GTP downstream pathways involved in Turner syndrome and neurodegenerative diseases. a RAP1 pathways regulating cell adhesion, migration, polarity, proliferation, survival and gene activation. DEGs in blue squares were down-regulated in 45,XO-hPGCLCs during specification from 45,XO-hiPSCs 2 in comparison with those of both 46,XX, and 46,XY. b The mutant G to V at site 12 is an activated form of RAP1. RAS, a conserved domain in RAS family. c Heat map of down-regulated genes from (a) in 45,XO-hiPSCs (n=3) and 45,XO-hPGCLCs (n=2). d Spot plots showing expression of representative genes in RAP1 pathway in 45,XO-hiPSCs (n=3) and 45,XO-hPGCLCs (n=2). Y-axis indicates expression levels (Log2 (FPKM+1)). *P < 0.05; **P < 0.01. [file 13578_2022_925_MOESM1_ESM.pdf]
